# ATP7B expression confers multidrug resistance through drug sequestration

**DOI:** 10.18632/oncotarget.8059

**Published:** 2016-03-14

**Authors:** F M Moinuddin, Yoshinari Shinsato, Masaharu Komatsu, Ryoichi Mitsuo, Kentaro Minami, Masatatsu Yamamoto, Kohich Kawahara, Hirofumi Hirano, Kazunori Arita, Tatsuhiko Furukawa

**Affiliations:** ^1^ Department of Neurosurgery, Kagoshima University Graduate School of Medical and Dental Sciences, 8-35-1 Sakuragaoka, Kagoshima 890-8544, Japan; ^2^ Department of Molecular Oncology, Kagoshima University Graduate School of Medical and Dental Sciences, 8-35-1 Sakuragaoka, Kagoshima 890-8544, Japan; ^3^ Center for the Research of Advanced Diagnosis and Therapy of Cancer, Graduate School of Medical and Dental Sciences, Kagoshima University, 8-35-1 Sakuragaoka, Kagoshima 890-8544, Japan; ^4^ Division of Food and Chemical Biology, Faculty of Fisheries, Kagoshima University, 4-50-20, Shimoarata, Kagoshima 890-0056, Japan

**Keywords:** ATP7B, copper transporter, multidrug resistance, doxorubicin, SN-38

## Abstract

We previously reported that ATP7B is involved in cisplatin resistance and ATP7A confers multidrug resistance (MDR) in cancer cells.

In this study, we show that ATP7B expressing cells also are resistant to doxorubicin, SN-38, etoposide, and paclitaxel as well as cisplatin.

In ATP7B expressing cells, doxorubicin relocated from the nuclei to the late-endosome at 4 hours after doxorubicin exposure. EGFP-ATP7B mainly colocalized with doxorubicin.

ATP7B has six metal binding sites (MBSs) in the N-terminal cytoplasmic region. To investigate the role of the MBSs of ATP7B in doxorubicin resistance, we used three mutant ATP7B (Cu0, Cu6 and M6C/S) expressing cells. Cu0 has no MBSs, Cu6 has only the sixth MBS and M6C/S carries CXXC to SXXS mutation in the sixth MBS. Cu6 expressing cells were less resistance to the anticancer agents than wild type ATP7B expressing cells, and had doxorubicin sequestration in the late-endosome. Cu0- and M6C/S-expressing cells were sensitive to doxorubicin. In these cells, doxorubicin did not relocalize to the late-endosome. EGFP-M6C/S mainly localized to the trans-Golgi network (TGN) even in the presence of copper. Thus the cysteine residues in the sixth MBS of ATP7B are essential for MDR phenotype.

Finally, we found that ammonium chloride and tamoxifen suppressed late endosomal sequestration of doxorubicin, thereby attenuating drug resistance. These results suggest that the sequestration depends on the acidity of the vesicles partly.

We here demonstrate that ATP7B confers MDR by facilitating nuclear drug efflux and late endosomal drug sequestration.

## INTRODUCTION

ATP7A and ATP7B are copper transporters existing in the Golgi membrane and share 67% amino acid identity. They have six metal binding sites (MBS) in the N-terminal cytoplasmic region and 8 membrane spanning segments. They have important roles in the copper homeostasis in animal cells [1–3]. Several mutations of ATP7A and ATP7B are causes of Menkes and Wilson disease respectively [4].

In the other hand, we previously found that high expression of ATP7B confers resistance against cisplatin and this has since been confirmed by other groups [5–7]. ATP7B expression has been reported as a poor prognostic marker in some cancers receiving cisplatin-base chemotherapy [8, 9]. ATP7A also has been reported to confer cisplatin resistance [10].

Additionally we indicated that ATP7A expressing cells are resistant against not only cisplatin but also a variety of anticancer agents including doxorubicin, SN-38, etoposide, vincristine and paclitaxel through vesicle transport dependent ways. Surgically resected ATP7A positive human colon tumor cells were significantly resistant to SN-38 than ATP7A negative cells *ex vivo* [11]. That means ATP7A confers multidrug resistance (MDR) of cancer cells and related to drug resistance in clinical cancer cells.

MDR is a characteristic that the cells are resistant to multiple structurally unrelated anticancer agents and an obstacle for successful cancer chemotherapy. MDR is caused by expression of some ABC transporters, modification of DNA repair systems, apoptosis defects and several more mechanisms [12, 13]. However there are no established therapies to overcome MDR in practice so far [14, 15].

Here we evaluated drug sensitivities of ATP7B expressing cells to make clear that ATP7B also confers MDR and examined the mechanism of ATP7B mediated drug resistance with mutant ATP7B expressing cells and imaging of doxorubicin localization.

## RESULTS

### Resistance to anticancer agents of ATP7B expressing cells

We previously reported that ATP7A confers resistance against a wide variety of anticancer agents in addition to cisplatin [11]. We first examined whether wt ATP7B expressing KB/WD cells are resistant to anticancer agents other than cisplatin by 3-(4, 5-dimethylthiazol-2-yl)-2, 5-diphenyltetrazolium bromide (MTT) assay. We found that KB/WD cells are significantly resistant to doxorubicin (17.25fold), SN-38 (38.14 fold), etoposide (25.84 fold), and paclitaxel (2.00 fold) as well as cisplatin (12.03fold) in comparison with KB/EV, pRC/CMV plasmid transfected KB-3-1 cells (Table 1).

**Table 1:** Chemosensitivity of wt and mutant ATP7B-expressing cells

Cells	KB/EV	KB/WD		KB/Cu6		KB/Cu0		KB/M6C/S	
Agent	IC_50_	IC_50_	RR	IC_50_	RR	IC_50_	RR	IC_50_	RR
cisplatin (μM)	1.52 ± 0.02	18.27 ± 1.15	12.03^Ɨ^	5.00 ± 0.24	3.30^Ɨ^	1.71 ± 0.20	1.13	1.71 ± 0.12	1.12
doxorubicin (μM)	0.25 ± 0.02	4.27 ± 0.53	17.25^Ɨ^	1.84 ± 0.19	7.43^Ɨ^	0.29 ± 0.08	1.16	0.23 ± 0.04	0.95
SN-38 (nM)	17.65 ± 0.60	673.07 ± 23.59	38.14^Ɨ^	101.32 ± 3.56	5.74^Ɨ^	23.53 ± 1.94	1.33^Ɨ^	24.83 ± 0.44	1.40^Ɨ^
etoposide (μM)	5.22 ±0.94	134.77±6.73	25.84^Ɨ^	113.27±1.55	21.72^Ɨ^	5.71±0.37	1.10	6.25±0.02	1.20
paclitaxel (nM)	10.45 ±1.60	20.93±1.44	2.00^Ɨ^	14.18±1.23	1.35^Ɨ^	8.67±0.20	0.83	11.57±0.43	1.10

IC_50_ values are means ± SDs from triplicate determinations with MTT assay.

RR (relative resistance) is a value that IC_50_ for KBWD, MCu6, MCu0, or M6c/s divided with IC_50_ for KB/EV cells, respectively.

Ɨ Indicates statistically significant (*p* <0.05)

### Doxorubicin localizes to the late endosome in ATP7B-expressing cells

**Figure 1:**
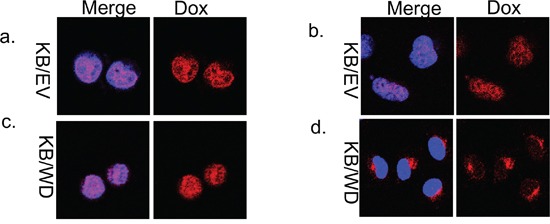
Doxorubicin localization in KB/EV and KB/WD cells Cells were exposed to doxorubicin for 1 hour and washed with PBS. Localization was observed in KB/EV cells at 0 time **a and b.** at 4 hours after washing PBS. Localization was observed in KB/WD cells **c.** at 0 time and **d.** 4 hours after washing PBS. Doxorubicin is red. The nuclei are stained with Hoechst 33342 (blue).

Doxorubicin has an intrinsic red fluorescence that allows observation of its localization. We observed the intracellular distribution of doxorubicin at 0 time and at 4 hours after doxorubicin treatment and washing with PBS with confocal laser fluorescence microscopy. In KB/EV cells, doxorubicin was localized predominantly to the nuclei at both time points (Figure 1a, 1b). Alternatively, in KB/WD cells, doxorubicin was localized in the nuclei at 0 time but after 4 hours, was predominantly localized in the cytoplasm (Figure 1c, 1d).

**Figure 2:**
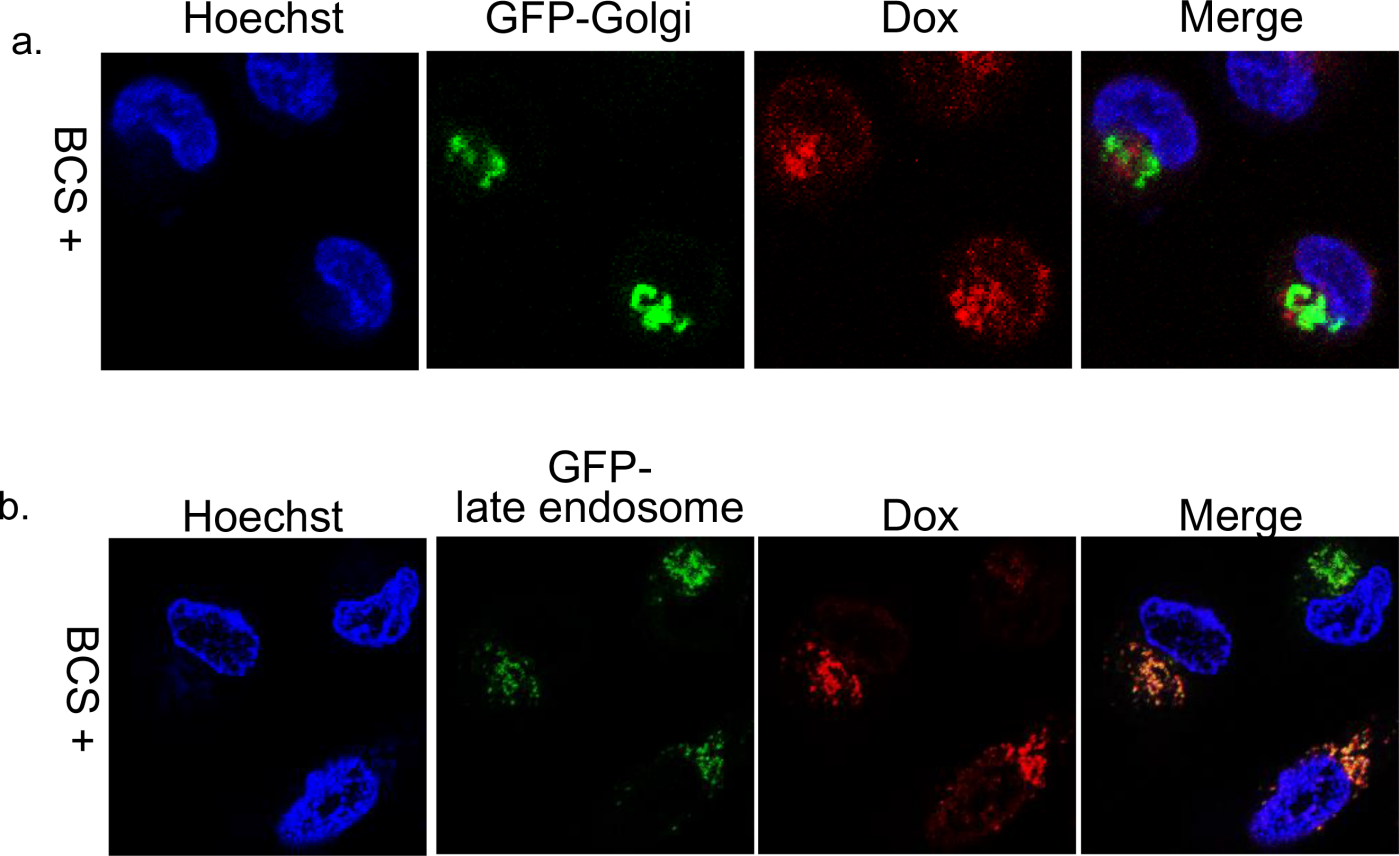
Subcellular localization of doxorubicin in KB/WD cells Subcellular localization of doxorubicin at 4 hours after its exposure and washing with PBS in **a.** GFP-Golgi (green) transfected KB/WD cells and **b.** GFP-late endosome (green) transfected KB/WD cells. Doxorubicin is red. The nuclei are stained with Hoechst 33342 (blue).

To determine the cytoplasmic localization of doxorubicin in ATP7B expressing cells, we observed doxorubicin distribution in the Green Fluorescence Protein (GFP)-Golgi or GFP-late endosome transfected cells in the presence of bathocuproine disulphonate (BCS), a copper chelate, at 4 hours after doxorubicin exposure. Doxorubicin in KB/WD cells predominantly co-localized with GFP-late endosomes and not with GFP-Golgi (Figure 2). Therefore, doxorubicin is sequestrated into the late endosome compartment from the nuclei in ATP7B expressing cells independent of copper.

### ATP7B localizes to the late endosome in the presence of doxorubicin

Not only copper but also cisplatin causes the relocalization of ATP7B from the trans-Golgi network (TGN) to the peripheral vesicles [6, 16]. We examined the localization of ATP7B after exposure of doxorubicin using EGFP-wt ATP7B transfection to KB-3-1 cells. When BCS is used to deplete copper, EGFP-ATP7B mainly colocalized with RFP-Golgi (Supplementary Figure 1a and 1b). By contrast in the presence of copper, EGFP-ATP7B localizes to the late endosome (Supplementary Figure 1c and 1d). These results are consistent with that copper levels influence the localization of ATP7B and result in its change from the TGN to the late endosome as previously reported [17, 18].

**Figure 3:**
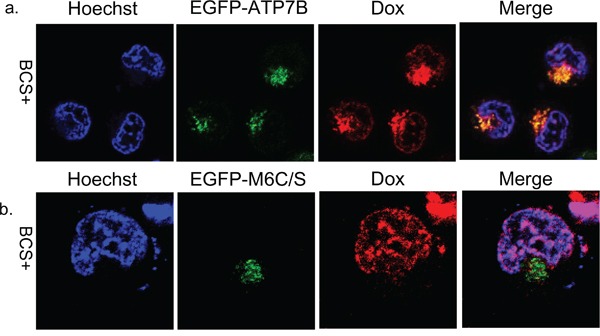
Subcellular localization of doxorubicin and EGFP-ATP7B or EGFP-M6C/S Subcellular localization of doxorubicin and ATP7B and M6C/S at 4 hours after doxorubicin exposure and washing with PBS in EGFP-ATP7B (green) **a.** or EGFP-M6C/S (green) **b.** transfected KB-3-1 cells. Doxorubicin is red. The nuclei are stained with Hoechst 33342 (blue).

Next, we examined the effect of doxorubicin on ATP7B localization in EGFP-ATP7B transiently transfected KB-3-1 cells in the presence of BCS. The ATP7B colocalized with doxorubicin in the late endosome at 4 hours after doxorubicin treatment (Figure 3a). Therefore, doxorubicin, copper, and cisplatin alter the ATP7B subcellular localization from the TGN to the late endosome [6, 16]. These results suggest that ATP7B can be activated with a metal unrelated chemical agent.

### The sixth metal binding site of ATP7B is critical for doxorubicin resistance and relocalization of ATP7B

In previous reports, the cytoplasmic MBSs, especially the cysteine residues in the sixth MBS (MBS6) of ATP7B are/is essential for copper transport and cisplatin resistance [16, 19]. We examined whether the MBSs in ATP7B are important for doxorubicin sequestration and resistance.

**Figure 4:**
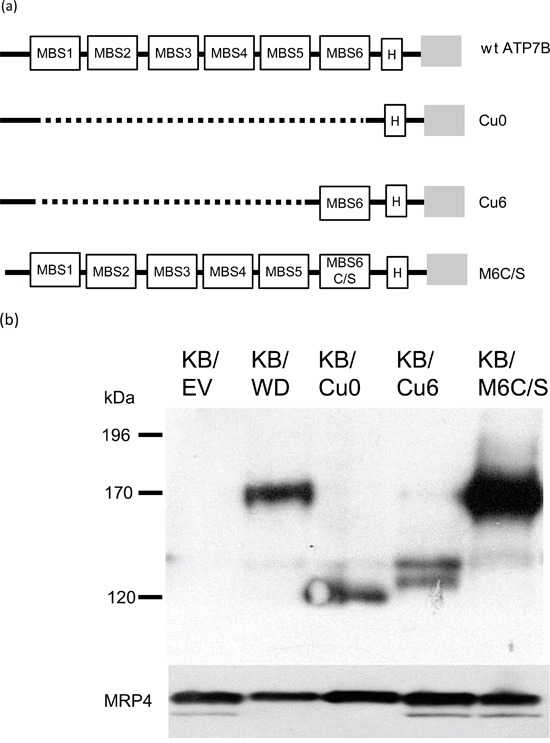
**a.** Schematic presentation of the N-terminal part of wt and mutant ATP7B proteins used in this study. Open boxes indicate MBSs, grey boxes indicate the transmembrane domain, black lines indicate protein, and grey dotted lines indicate deletions. H means HA epitope. MBS6C/S indicates that CXXC in the 6th MBS has been converted to SXXS. **b.** Expression of the wt and mutant ATP7B proteins from the indicated cells lysate detected by immunoblotting. ABCC4/MRP4 protein was indicated as loading control of membrane fraction protein.

Figure 4a presents a schematic drawing of the N-terminus of ATP7B proteins used in this study as described in the material and method. Cu0 has no MBSs, Cu6 has only the sixth MBS and M6C/S carries CXXC to SXXS mutation in the sixth MBS. Cu0, Cu6 and M6C/S expressing KB-3-1 cells are named KB/Cu0, KB/Cu6 and KB/M6C/S respectively.

The protein expression of wild type (wt) and mutant ATP7B was examined by immunoblotting using anti-HA antibody (Figure 1b). The expression of each ATP7B protein was comparable in each cell line. Full-length wt ATP7B and M6C/S (170 kDa) and the ATP7B deletion mutants, Cu0 and Cu6, were detected at their expected sizes 120 kDa and 125 kDa respectively (Figure 4b).

In MTT assay, KB/Cu0 cells were sensitive to the agents, suggesting that the cytoplasmic MBSs are important for resistance to doxorubicin and other anticancer agents. KB/Cu6 cells were more resistant to cisplatin (3.30-fold), doxorubicin (7.4-fold), SN-38 (5.7-fold), etoposide (21.7-fold), and paclitaxel (1.35-fold) than KB/EV cells, but less resistant than KB/WD wt ATP7B-expressing cells. KB/M6C/S cells were sensitive to these agents (Table 1). These results suggest that MBS6 of ATP7B is critical for doxorubicin resistance while MBSs 1-5 increase resistance but are not required.

**Figure 5:**
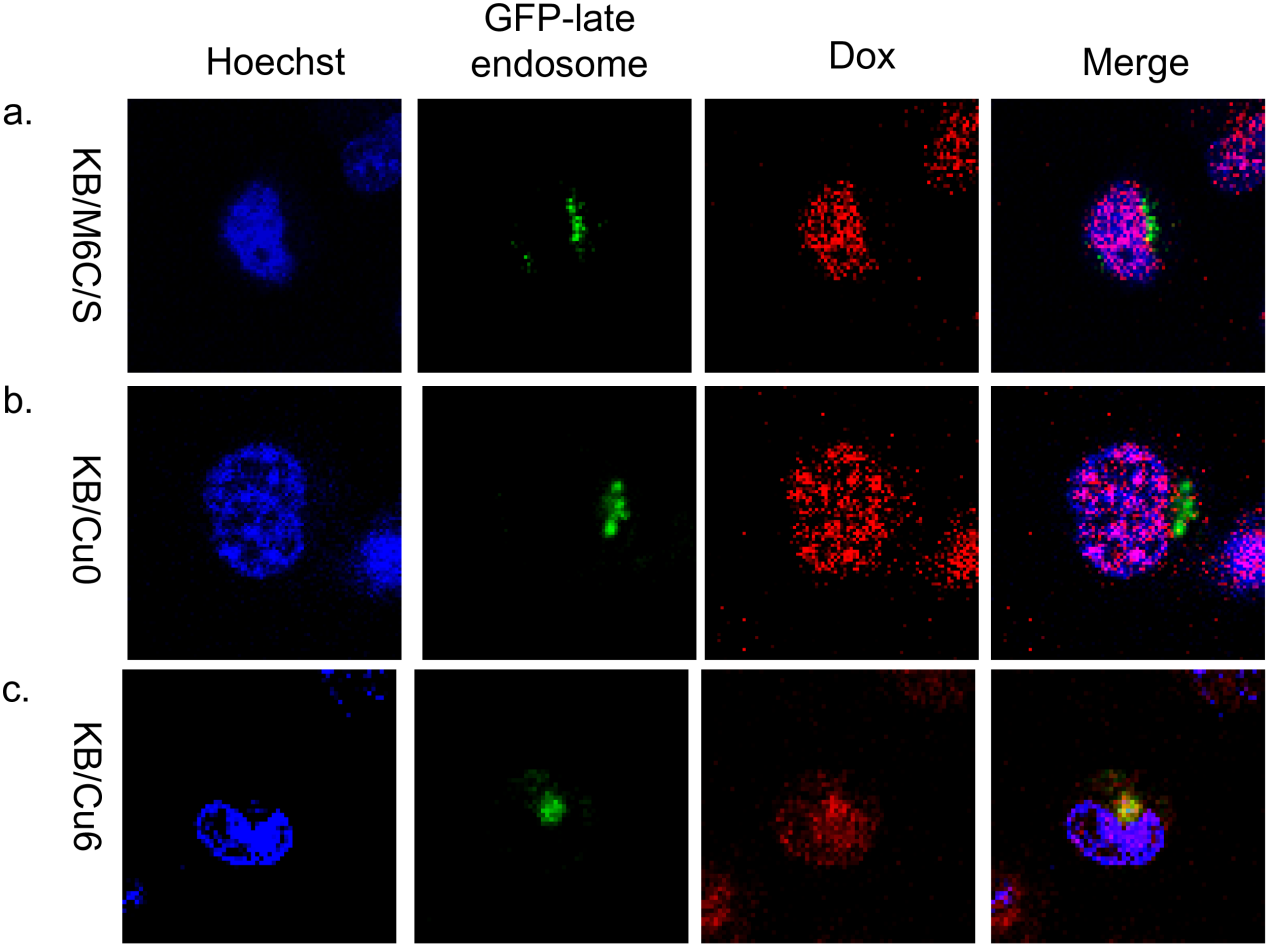
Subcellular localization of doxorubicin in mutant ATP7B expressing cells Subcellular localization of doxorubicin at 4 hours after doxorubicin exposure and washing with PBS in **a.** KB/M6C/S, **b.** KB/Cu0, and **c.** KB/Cu6 cells. GFP-late endosomes are green, doxorubicin is red. The nuclei are stained with Hoechst 33342 (blue).

To identify where doxorubicin localizes in these mutant ATP7B expressing cells, we observed doxorubicin localization after transfection with GFP-late endosome. In KB/Cu6 cells, which have some drug resistance, doxorubicin was detected in both the cytoplasm and nuclei at 4 hours after exposure and was partly co-localized with the GFP-late endosome like KB/WD (Figure 5c). In KB/M6C/S and KB/Cu0 cells, doxorubicin was mainly localized in the nuclei (Figure 5a, 5b). Furthermore in EGFP-M6C/S expressing KB-3-1 cells, doxorubicin localized to the nuclei and EGFP-M6C/S protein localized to cytoplasm differently from those in the EFGP-ATP7B expressing cells (Figure 3).

Therefore, the cysteine residues of MBS6 are essential for ATP7B mediated drug sequestration in the late endosome. These results support that the sequestration of doxorubicin is mediated by ATP7B through its interaction with doxorubicin via MBS6.

### Acidic condition in the vesicles contribute to doxorubicin sequestration

**Figure 6:**
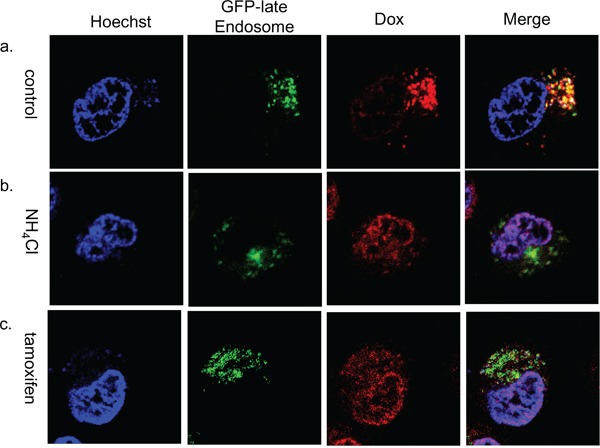
Subcellular localization of doxorubicin in NH_4_Cl or tamoxifen treated KB/WD cells Subcellular localization of doxorubicin in **a.** GFP-late endosome (green) transfected KB/WD cells at 4 hours after exposure, **b.** in the presence of 10 mM NH_4_Cl, or **c.** in the presence of 10 μM tamoxifen. Doxorubicin is red. The nuclei are stained with Hoechst 33342 (blue).

**Figure 7:**
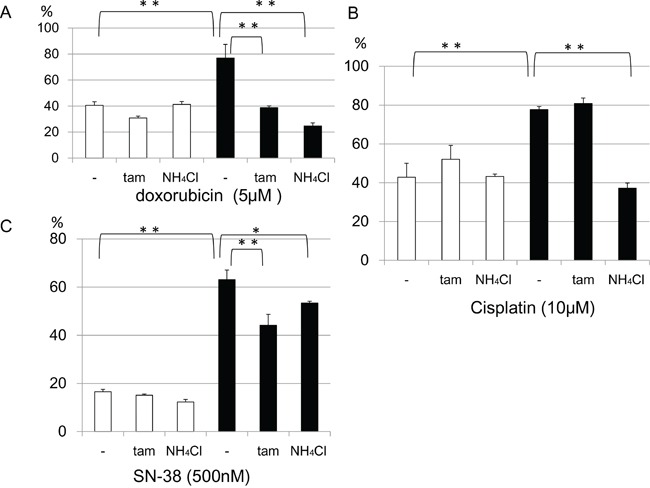
Effects of NH_4_Cl or tamoxifen (tam) on KB/EV and KB/WD cell viability in the presence of anticancer agents In the presence of **a.** 5μM doxorubicin, **b.** 10μM cisplatin, or **c.** 500nM SN-38, the effect of 10mM NH_4_Cl or10 μM tamoxifen on cell viabilities of KB/EV cells (open bar) and KB/WD cells (black bars) are indicated. Data represent means of triplicate determination.*P < 0.05, **P<0.001)

To investigate whether doxorubicin sequestration in the late endosome depends on the acidity of the vesicles, we examined doxorubicin localization in the presence of NH_4_Cl or tamoxifen. NH_4_Cl is a lysosomotropic agent that inhibits sequestration of weakly basic molecules in the vesicle by increasing the endo-lysosomal pH. Tamoxifen inhibits acidification of endosomes and lysosomes without increasing cytoplasmic pH [20, 21]. In the presence of NH_4_Cl or tamoxifen, doxorubicin accumulated in the nuclei and only slightly colocalized with the GFP-late endosome (Figure 6). Next, we estimated whether NH_4_Cl or tamoxifen could modify cell viability. The viability of doxorubicin treated KB/WD cells significantly decreased in the presence of both NH_4_Cl and tamoxifen, although there was no remarkable change in that of KB/EV cells. Preventing sequestration of drugs in the acidic vesicle by NH_4_Cl could increase the sensitivity of KB/WD cells against doxorubicin and SN-38 (Figure 7).

In addition to NH_4_Cl, tamoxifen can reduce ATP7B mediated drug resistance to doxorubicin, cisplatin, and SN-38. However, the effects of cisplatin are weaker than those of doxorubicin. This result is consistent with doxorubicin being a weak base (pKa 9.53/8.94 strongest acidic and strongest basic respectively), which allows it to accumulate in the acidic vesicles, while cisplatin (pKa 5.06) and SN-38 (pKa 9.68/3.91 strongest acidic and strongest basic respectively) are more acidic and acidification has weaker effect to accumulation of these compounds. These results indicate that ATP7B mediated doxorubicin sequestration partially depends on the acidity of the vesicles.

## DISCUSSION

We previously reported that expression of either ATP7B confers cisplatin resistance and that the expression of ATP7A confers resistance to several structurally unrelated anticancer drugs including cisplatin [5, 11]. The involvement of ATP7B in resistance to other anticancer drugs has not been investigated. Here we show that ATP7B confers resistance to doxorubicin, SN-38, etoposide, and paclitaxel, making ATP7B another candidate of MDR.

ATP7B normally resides in the TGN of hepatocytes. In the presence of elevated copper levels, ATP7B is redistributed from the TGN to the late endosome/lysosome compartments [17, 18]. Tagged wt ATP7B localizes to the TGN under both copper- and cisplatin-free conditions and is dispersed throughout the cell after 1 hour of exposure to either cisplatin or copper [6, 16]. Fluorescein-labeled cisplatin localizes to the TGN and lysosome and colocalizes with both ATP7B and ATP7A [6, 22]. Here we show that exposure to doxorubicin also induces redistribution of ATP7B to the late endosome, similar to exposure to copper and cisplatin. Additionally, we show ATP7B facilitates efflux from the nuclei and sequestration of doxorubicin to the late-endosome. Taken together, these results suggest that ATP7B is activated by doxorubicin as well as by copper and cisplatin.

The CXXC motif in MBS6 of ATP7B is essential for the transport of Cu and for trafficking in response to cisplatin [16, 23, 24]. KB/M6C/S cells are sensitive to cisplatin, doxorubicin, SN-38, etoposide and paclitaxel suggesting that the CXXC motif of MBS6 is important for resistance to these anticancer agents. Alternatively, KB/Cu6 cells are resistance to these agents, but are less resistant than KB/WD cells to them. The resistance of the mutant-expressing cells to doxorubicin is correlated with the amount of doxorubicin accumulation in the late endosome based on our confocal microscopic study (Figure 4 and 5). Thus, MBS6 is critical to drug resistance; however, MBS6 alone is not sufficient to confer resistance comparable to wt ATP7B. MBSs 1-5 have supportive roles in the function of ATP7B.

Our results are compatible with previous studies investigating the MBSs of ATP7B. The second MBS in the N-terminal domain of ATP7B is important in receiving copper from Atox1, a cytosolic copper chaperone [25]. Platinum can also transfer copper to the second MBS from Atox1 [26]. The fragment of N-terminal ATP7B including MBSs 1-4 binds with the copper-bound Atox1 in the yeast two hybrid system [27]. Cisplatin also binds to MBSs 1-4 but not MBSs 5 or 6 of ATP7B based on studies using proteins expressed in *Escherichia coli* [28]. Therefore, MBSs 1-4 might have higher affinity to the copper than MBS6. These reports are consistent with MBSs 1-4 having a supportive function of ATP7B transport activity. MBSs 1-5 is expected to also interact with doxorubicin since KB/Cu6 cells are less resistant to anticancer agents in comparison with KB/WD cells.

Cisplatin was shown to be transported by the ATP7B-expressing vesicle using the baculovirus expressing system [24]. Recently, electrical measurements using a membrane fraction of ATP7A- or ATP7B-expressing Cos-1 cells demonstrated a charge transfer in the presence of cisplatin, indicating an ATP-dependent vectorial displacement of a charged cisplatin complex by ATP7A and ATP7B [29]. We previously showed that SN-38 could be transported into the membrane vesicles of ATP7A-expressing cells [11]. These results suggest ATP7A and ATP7B directly transfer cisplatin or SN-38 to acidic vesicles.

Since many chemotherapeutic agents, which target molecules including nucleic acids, topoisomerases, and DNA polymerase, localize to the nucleus, alteration of the intracellular distribution of these chemicals strongly influences their efficacy [30–32]. Vesicle acidification has been proposed to play one of major roles in drug sequestration [33]. Previously, we found that in ATP7A-expressing cells, monensin, which disturbs the acidity of the vesicles, could relocate doxorubicin from intracellular vesicles to the nuclei [11]. In our present study, the inhibition of vesicle acidification with NH_4_Cl or tamoxifen altered drug distribution and significantly reversed the cell viability in ATP7B expressing cells. Thus, ATP7B confers resistance through late endosomal sequestration, which partly depends on acidification.

In this study indicates that ATP7B potentially facilitate doxorubicin efflux from the nuclei and following late endo-lysosomal drug sequestration, and confers drug resistance to cancer cells. More detailed analysis of the mechanism of drugs sequestration is required.

Appearance of drug resistant, especially MDR, cells hinders successful chemotherapy treatments. Several mechanisms of MDR, including expression of some ABC transporters, increase detoxification, DNA repair and apoptosis defects have been reported [12, 13]. The therapy against MDR is not established, one of the reason is not all mechanisms of MDR have not been elucidated [14]. It has been reported that ATP7B expression elevate in several human malignancies, including ovarian, gastric, and breast cancers when compared with non-cancer tissues and its high expression is a poor prognostic marker in ovarian and oral squamous cell cancers treated with cisplatin-base chemotherapy [8, 34–36]. Using PrognoScan (http://www.abren.net/PrognoScan/) we found that high ATP7B expression is a poor prognosis index in several neoplasms which non-platina base chemotherapy are applied as standard protocol for: AML (data set GSE8970: P<0.014613), DLBCL (data set E-TABM-346: p<0.026222), follicular lymphoma (data set GSE16131-GPL97: p<0.040192), glioma (data set GSE4412-GPL97: P<0.005900).

According to our analysis and previous reports, ATP7B is expected to be important position in MDR of human cancers clinically as well as cisplatin resistance. In this study, we show that ATP7B confers MDR to cancer cells, similarly to ATP7A, by facilitating nuclear efflux and following late endosome drug sequestration.

## MATERIALS AND METHODS

### Chemicals

We purchased G-418, cisplatin, doxorubicin etoposide, paclitaxel and tamoxifen form Sigma Chemical Co., (St, Louis, MO), Glutamine, NH_4_Cl and CuCl_2_ from Wako (Osaka, Japan), and HEPES and BCS from Dojindo (Kumamoto, Japan). SN-38 was obtained by donation (Daiichi Sankyo Pharmaceutical Co. Ltd.).

### Cell culture and cells

All cells were cultured in DMEM (Nissui Pharmaceutical Co. Ltd., Tokyo, Japan) containing 10% fetal calf serum (Thermo Scientific, Logan, UT), 10 mM HEPES (7.5 pH), 2 mM L-glutamine and 100 units/ml penicillin in 5% CO_2_ at 37°C. Wild type (wt) ATP7B expressing cells named KB/WD cells were established by wt ATP7B transfection to human epidermoid carcinoma, KB-3-1 cells as described previously [5]. KB/EV cells were established from KB-3-1 cells transfected with pRc/CMV.

### Construction of mutant ATP7B cDNAs and transfection

Wt ATP7B and two mutant ATP7B, Cu0 (deleted all MBS) and Cu6 (deleted the 1st-5th MBSs, MBS1-5) plasmids were kindly presented by Drs. Sugiyama and Terada (Akita University) Cu0 and Cu6 were constructed as described previously [37]. Another ATP7B mutant M6C/S, the cysteines of which were replaced with serines in MBS6, was generated using site-directed mutagenesis with forward primer: 5′ CCTCTGTCCACAACATAGAGTCCA 3′ and reverse primer: 5′ AAGCGCTGGTCATCCCTGTGATTG 3′. These cDNAs were ligated into pRc/CMV (Thermo Fisher Science, Waltham, MA) and transfected into KB-3-1 cells using Lipofectamine 2000 (Thermo Fisher Science) with G418 selection as described previously [5]. Each single clone that expresses comparative amounts of ATP7B protein to that in KB/WD cells was used in the following analysis.

To produce EGFP-wt ATP7B and EGFP-ATP7B M6C/S cDNA, *BamH*1-*Xba*1 fragments that include the entire coding region of pRc/CMV-wt ATP7B and M6C/S cDNA were ligated into the *Bgl* II and *Xba*I sites of pEGFP-C2 vector (Clontech, Japan). These plasmids were transiently transfected into KB-3-1 cells with Lipofectamine 2000 and observed two days after transfection.

### Preparation of membrane protein

Membrane proteins were prepared as described previously [5]. Protein concentration was determined by Bio-Rad Protein Assay Kit according to the manufacturer's protocol (Bio-Rad Laboratories, Hercules, CA).

### Immunoblotting

Membrane fraction protein (100μg) was subjected to 7.5% SDS-PAGE under reducing conditions. Immunoblotting was carried out as described previously [38]. A polyclonal antibody against the HA epitope (Santa Cruz Biotechnology, Santa Cruz, CA) or anti-ABCC4/MRP4 monoclonal antibody (M4I-10, Monsan, Netherlands) was used as a primary antibody, while horseradish peroxidase conjugated anti-rabbit or anti-mouse IgG (GE Healthcare, Buckinghamshire, England) was the secondary antibody respectively. Immunoreactive bands were visualized with enhanced chemiluminescence using ECL prime western blotting system (GE Healthcare).

### MTT assay

Chemosensitivity was estimated by MTT colorimetric assay. We plated 3 × 10^3^ cells/well of KB/EV, KB/Cu0, KB/Cu6, and KB/M6C/S or 5 × 10^3^ cells/well of KB/WD into 96-well plates and performed MTT assay as described previously [5].

### Subcellular distribution of doxorubicin

We plated all cell lines at a density of 2 × 10^4^ cells/well on a four-compartment glass-bottom 35-mm dish (Greiner Bio One, Frickenhausen, Germany) and the cells were allowed to attach overnight. The cells were exposed for 1 hour to 5 μM doxorubicin at 37°C. To verify the intracellular localization of doxorubicin, cells were transfected with GFP-Golgi, RFP-Golgi, GFP-late endosome, or RFP-late endosome (Cell Light Reagents BacMam 2.0, Thermo Fisher) according to the manufacturer's protocol. Following 5 μM doxorubicin exposure for 1 hour, cells were incubated in DMEM with or without 200 μM BCS, 10 mM NH_4_Cl, or 10 μM tamoxifen for 4 hours. All cells were stained with Hoechst 33342 (Thermo Fisher) in PBS (1:2000) for 5 minutes, washed with PBS three times, and observed using a confocal laser microscopy (Zeiss LSM700, OberKochen, Germany).

### Cell viability assay

To determine the effects of NH_4_Cl and tamoxifen on cell viability, cells were seeded at 2000 cells/well in 96 well plates. After an overnight incubation, cells were treated with doxorubicin, SN-38 and cisplatin with or without 10 mM NH_4_Cl or 10 μM tamoxifen for 24 hours. The cell viability was determined using the Cell Titer-Glo Luminescent Cell Viability Assay Kit (Promega, Madison, WI) according to the manufacturer's instructions. Luminescence was measured using Fluoroskan Ascent FL (Thermo Fisher).

### Statistical analysis

Differences between groups were analyzed by two-tailed Student's *t* test. *P* < 0.05 was considered significant.

## SUPPLEMENTARY FIGURE


