# Supplementary material for: ATP7B expression confers multidrug resistance through drug sequestration
Source: Oncotarget. 2016 Mar 14;7(16):22779–90. doi: 10.18632/oncotarget.8059 (PMC5008400; doi:10.18632/oncotarget.8059)
Supplement: Supplementary file 1 [file oncotarget-07-22779-s001.pdf]

SUPPLEMENTARY FIGURE

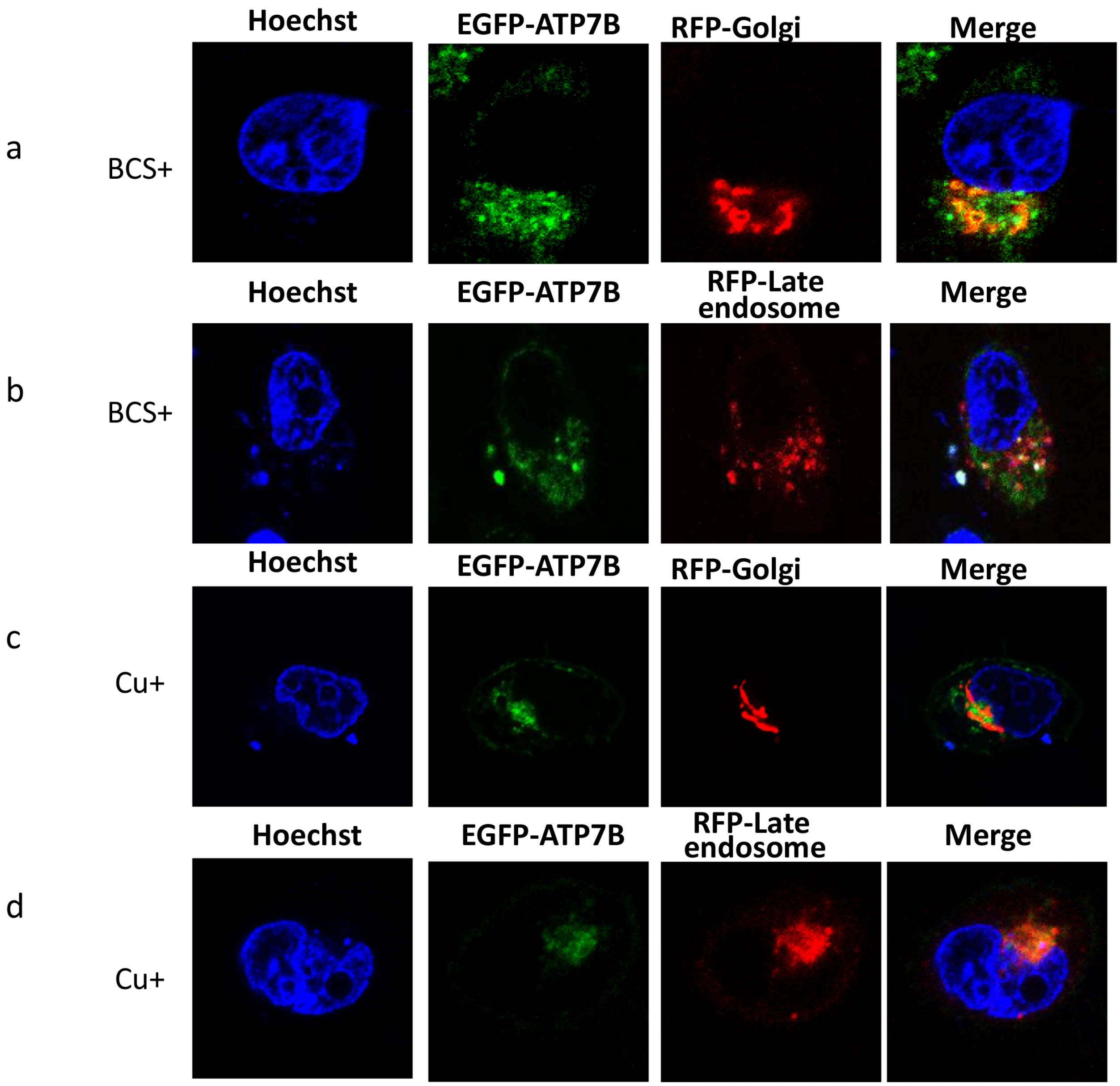

Supplementary Figure S1: Subcellular localization of EGFP-ATP7B (green) in RFP-Golgi (red) transfected KB-3-1 cells. a. in the presence of 200 µM BCS or **c.** after incubation with 10 µM $CuCl_2$ after exposure to 200 µM BCS and a wash with PBS. Subcellular localization of EGFP-ATP7B (green) in RFP-late endosome (red) transfected cells **b.** in the presence of 200 µM BCS treated **d.** at 4 hours after incubation with 10 µM $CuCl_2$ after exposure to 200 µM BCS and a wash with PBS. The nuclei are stained with Hoechst 33342 (blue).
